# Supplementary material for: Chromothripsis during telomere crisis is independent of NHEJ, and consistent with a replicative origin
Source: Genome Res. 2019 May;29(5):737–49. doi: 10.1101/gr.240705.118 (PMC6499312; doi:10.1101/gr.240705.118)
Supplement: Supplemental Material [file supp_gr.240705.118_Supplemental_file_1.zip › contigs/annotated_contigs/DB54/contig.3.DB54_length_874_mean_cov_9.52173913043.docx]

**DB54_length_874_mean_cov_9.52173913043**

```
CCAAAATTAAGGTATATGCACATGAAAGGAACTTGCTTATAAGGCATTATCAAGTGCATATGTGTTACCACCACGATTTGTTTTTATCA
 >chr1:193315902-193316354 + E=5e-254
TGATGTTCATAACTATCTGGCATATATGTGTTTATTTGTTTGCTTGTTATTTGTGTCCTCCCTTTGGATGGTAACTTTTATAAGGAAAA

ATATGCATCTTATGAATCTATATTCCATCAGTTAGAGCAGTGTCAGGTACACAGTGGCTGTTAGATGTTGGCTAAACAAATGGATAAAT

GAGTCATCTTAGGCAGATATTTTTCTGCAGTGAAATTGTGCCCTGTCTTGACTTTGCAGAAATTTCTCACCACTGTTTGGAGATATACA

CCTGTATGTCCAACTGCCTGCTTGACTTTTCCACTTGAATGTCTAGCAGGAATCTCACATGTCCACAATATTCCTCCTTATTTTTCAAA

CCAAG|TC|AATTTCATTCTCTGTGACTAGTAGTATCAAGTTATCTCTATCCCTGAGAGAGAGACAAAGGTCAGTGCTAAAATAGTTGA
      >chr1:193315256-193315476 - E=2e-120
GTCTGTGAGTGAGAGAAGTCATAAAAGGTCTCAAATCAACCTTGCCAGCTGTTTTTCCAAAGTCAAAGAAGAAAGTGAGAGGAAAACCA

ATAATCTGGAAGTTGATACAAATCTGATCCTAACATTTCATCTACCTCA|TGT|CTGTAATTCCAGCACTTTGGGAGGCCAAGGCGGGC
                                                     >chr8:71476524-71476719 - E=2e-78
AGATCACGGGGTCAGGAGATCGAGACCATCCTGGCTAACACAGTGAAACCCCATCTCTACTAAAAATACAAAAAATTAGCCGGGCGTGG

TGGCAGGCGCCTGTAATCCCAGCTACTCGGGAGGCTGAGGCAGGAGAATTGCTTGAACCCAGGAGACAGAG|GATGCA
```
